# Invasive Strategy in Octogenarians with Non-ST-Segment Elevation Acute Myocardial Infarction

**DOI:** 10.31083/j.rcm2503078

**Published:** 2024-02-28

**Authors:** Sara Álvarez-Zaballos, Miriam Juárez-Fernández, Manuel Martínez-Sellés

**Affiliations:** ^1^Department of Cardiology, Hospital General Universitario Gregorio Marañón, 28007 Madrid, Spain; ^2^Medicine Department, Universidad Europea, 28670 Madrid, Spain; ^3^Medicine Department, Universidad Complutense, 28005 Madrid, Spain

**Keywords:** acute coronary syndrome, octogenarians, NSTEMI, frailty

## Abstract

With population aging and the subsequent accumulation of cardiovascular risk factors, a growing proportion of patients presenting with acute coronary syndrome (ACS) are octogenarian (aged between 80 and 89). The marked heterogeneity of this population is due to several factors like age, comorbidities, frailty, and other geriatric conditions. All these variables have a strong impact on outcomes. In addition, a high prevalence of multivessel disease, complex coronary anatomies, and peripheral arterial disease, increases the risk of invasive procedures in these patients. In advanced age, the type and duration of antithrombotic therapy need to be individualized according to bleeding risk. Although an invasive strategy for non-ST-segment elevation acute myocardial infarction (NSTEMI) is recommended for the general population, its need is not so clear in octogenarians. For instance, although frail patients could benefit from revascularization, their higher risk of complications might change the risk/benefit ratio. Age alone should not be the main factor to consider when deciding the type of strategy. The risk of futility needs to be taken into account and identification of risk factors for adverse outcomes, such as renal impairment, could help in the decision-making process. Finally, an initially selected conservative strategy should be open to a change to invasive management depending on the clinical course (recurrent angina, ventricular arrhythmias, heart failure). Further evidence, ideally from prospective randomized clinical trials is urgent, as the population keeps growing.

## 1. Introduction 

**Fig. 1.**
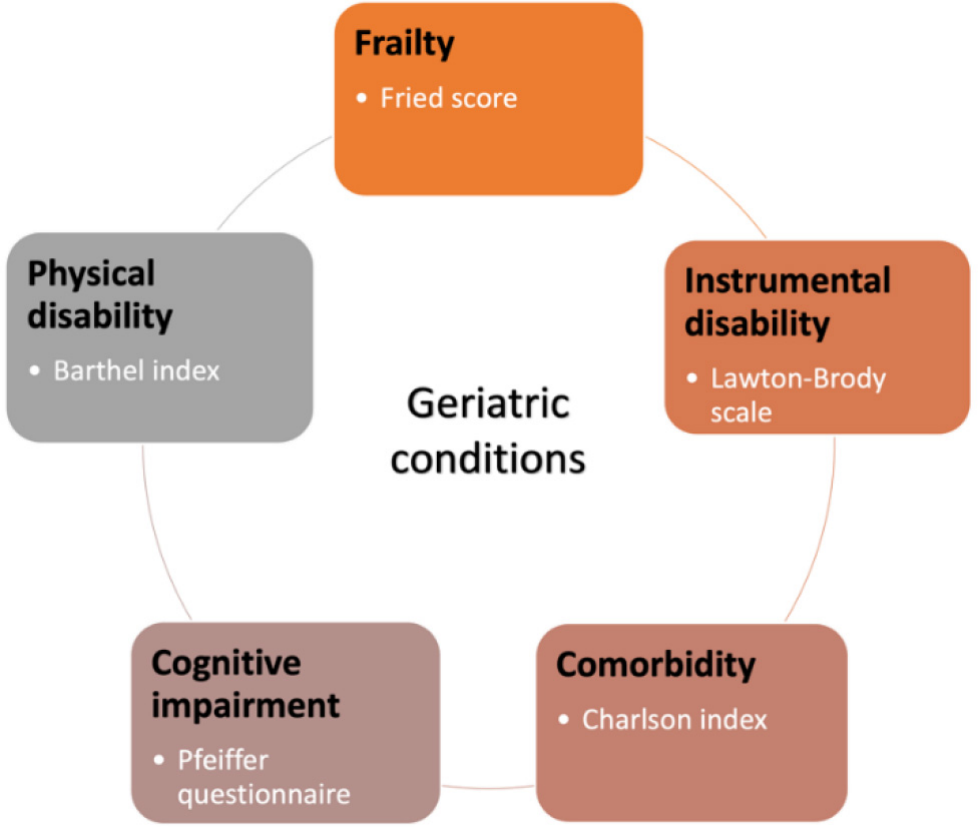
**Geriatric conditions**. Common geriatric conditions and some simple indexes to assess them.

The prevalence of coronary artery disease increases with age. Acute coronary syndromes (ACS) are common in octogenarians (aged between 80 and 89) and age is associated with a poor prognosis [1]. Elderly patients have been underrepresented in ACS clinical trials, particularly in the case of frail patients and those >80 years [2]. Population aging, and the subsequent accumulation of cardiovascular risk factors make ACS in octogenarians increasingly common [3]. Although improvements in percutaneous coronary interventions, such as radial access and drug eluting stents, have minimized the risks of an invasive strategy in octogenarians with non-ST-segment elevation acute myocardial infarction (NSTEMI), the benefits and risks of an invasive strategy in this population are still unclear [4]. In this review, we aim to focus on the peculiarities of invasive strategy for octogenarians, taking in consideration age, comorbidities, frailty, and other geriatric conditions (Fig. 1). We will show how the marked heterogeneity of octogenarians can influence management and outcomes.

## 2. Age

Cardiovascular changes associated with aging can predispose an individual to coronary artery disease, myocardial ischemia and ACS. The increased stiffness of the aorta and main arteries, frequently seen in the elderly, leads to increased resistance of left ventricular ejection and main systolic blood pressure, with a decrease in diastolic pressure. This causes a compensatory left ventricular hypertrophy, with increased myocardial work and oxygen demand, whereas the loss in diastolic pressure diminishes coronary perfusion pressure, leading to an imbalance in myocardial oxygen demand and supply, which predisposes older adults to type 2 myocardial infarction and NSTEMI. In addition, the combination of endothelial dysfunction and a state of chronic inflammation that is seen in older patients begets atherosclerosis, contributing to coronary artery disease development and progression [5].

NSTEMI clinical presentation in advanced-age patients is frequently atypical. Dyspnea, or even syncope or malaise might be present [6]. The frequent presence of advanced and complex coronary artery disease and comorbidities favor complications of interventional procedures [7].

**Fig. 2.**
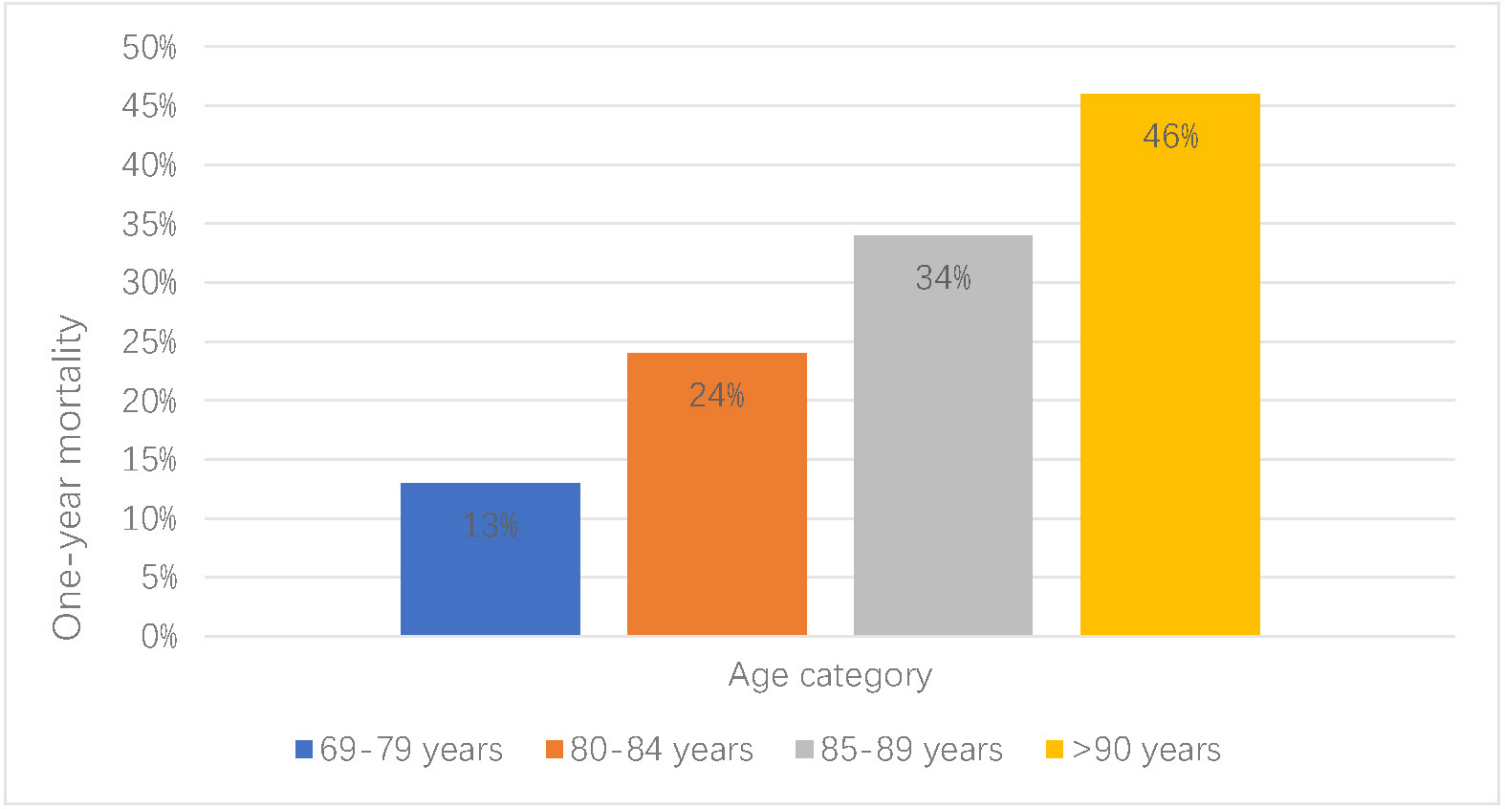
**Mortality of acute coronary syndromes (ACS) in the elderly**. One-year mortality of acute coronary syndrome increases with age.

Age is related to ACS in hospital and long-term mortality [3, 8]. In the Randomized Intervention Trial of unstable Angina 3 (RITA-3 trial), age was the strongest predictor of death or myocardial infarction, with more than a doubling risk for each 10 years of age over 60 years [9]. In-hospital mortality for elderly patients with ACS ranges between 8–11% [10, 11]. One-year mortality increases with age (Fig. 2) [12]. High mortalities after percutaneous coronary intervention in patients >85 years have been described [13], and mortality is particularly high in nonagenarians (aged between 90 to 99) [14, 15], that, compared with octogenarians, have increased cardiovascular event rates and higher risk of all-cause mortality [15, 16]. Few studies have specifically address centenarians with ACS. The Can Rapid Risk Stratification of Unstable Angina Patients Suppress Adverse Outcomes with Early Implementation of the ACC/AHA (American College of Cardiology/American Heart Association) Guidelines (CRUSADE) registry showed a poor prognosis for nonagenarians and even more so for centenarians [17] and data form the Polish Registry of ACS, including 104 centenarians with myocardial infarction, found an in-hospital mortality of nearly 35% and one-year mortality of 70% [18].

## 3. Comorbidities

Multimorbidity is present in most elderly patients with ACS and can interfere with treatment. Comorbidities can be assessed using the Charlson Comorbidity Index [19], which includes up to 19 conditions. A simplified version with six comorbidities has been proposed for a more accurate assessment of comorbidity in this specific population [20]. They include renal failure, anemia, diabetes mellitus, cerebrovascular disease, peripheral artery disease, and chronic lung disease. Mortality increased with a higher number of comorbidities [20]. Evidence suggests that the benefits or revascularization in older patients diminish with more comorbidity burden [21].

Chronic kidney disease is more prevalent in older patients because of the progressive loss of kidney function with age [22]. Cardiorrenal syndrome can also play a role in the severity of kidney injury, which is associated with worse outcomes [23, 24]. The Impacto de la fragiLidad y Otros síndromes GEriátricos en el manejo y pronóstico Vital del ancianO con Síndrome Coronario Agudo sin elevación de segmento ST (LONGEVO-SCA) registry studied consecutive patients aged ≥80 years and assesed the impact of renal function in outcomes such as mortality or readmissions and its relation to frailty status. A significantly higher incidence of outcomes was observed with renal disease. However, this association was not significant in frail patients, probably due to the impaired prognosis of frailty *per se * [25].

Diabetes is known to be associated with mortality in patients with ACS [26]. In the Elderly-ACS 2 trial, diabetes was associated with an increase in cardiovascular mortality but only due to its association with higher rates of comorbidities and cardiovascular risk profile [27]. In the elderly, diabetes seems to be associated with a higher incidence of events mainly among frail patients, with a similar incidence of mortality or readmission in non-frail patients [28, 29]. The impact of diabetes on prognosis seems to be particularly strong in elderly women [30].

Anemia is a strong predictor of prognosis in ACS, with an increased risk of mortality for hemoglobin levels below 11 g/dL [31]. It is also a predictor of bleeding in older patients undergoing percutaneous coronary intervention, non-inferior than well-known bleeding scores like PREdicting bleeding Complications In patients undergoing Stent implantation and subsEquent Dual Anti Platelet Therapy (PRECISE-DAPT) or CRUSADE [32]. The association between anemia and mortality seems to be more significant in robust patients, whereas frailty leads to a poorer prognosis irrespective of hemoglobin levels [33].

Cognitive impairment is prevalent in the elderly (>25% in patients >80 years) and is associated with risk of dementia [34]. Other related conditions such as Alzheimer’s disease and vascular cognitive impairment also increase with age [2]. A stressful event such as an ACS can worsen cognitive function in older patients and contribute to further deterioration [2].

## 4. Frailty

Frailty is defined as a clinical syndrome, characterized by a loss of biological reserves, which can lead to failure of the mechanisms of homeostasis after a stressor event [35]. Two main models of frailty have been described: the phenotype model defines frailty as the presence of three or more signs and symptoms, such as weight loss, self-reported exhaustion, weakness, slow walking speed, and low physical activity [36]. The cumulative model includes comorbidities and calculates a frailty score according to the number of “deficits” present, which can be symptoms, signs, diseases, or disabilities [37, 38]. The prevalence of frailty is up to 22% in those aged >80 years and is higher in women than in men [39, 40].

Frailty is independently associated with mortality in advanced-age patients with ACS [41, 42, 43, 44, 45]. In the LONGEVO-SCA registry [46] the presence of pre-frailty and frailty increased six-month mortality 2.7 and 3.0 times respectively [47] and the association remained significant in long-term follow-up [48]. In addition, frailty is a risk factor for the development of other geriatric syndromes [40].

The prevalence of frailty in the elderly is twice as high in women than in men, and elderly women have a higher incidence of comorbidities, accounting for the increased mortality of frail women after an ACS [49]. In LONGEVO-SCA, the female sex was an independent predictor of death/hospitalization, and frailty was associated with higher mortality in women, but not prefrailty [45].

## 5. Other Geriatric Conditions and Comprehensive Geriatric Assessment

Functional and sensorial decline, delirium, falls, and polypharmacy are also common in the elderly. The presence of geriatric syndromes may affect the clinical course, prognosis and treatment [2]. Nutritional status is also related to outcomes in advanced-age patients with ACS and the Mini Nutritional Assessment-Short Form (MNA-SF) has been shown to be an independent risk factor for mortality in this population [50].

Delirium is a fluctuating state of confusion with alterations in attention and cognitive function. Its incidence depends on the setting of the population, reaching up to 20% in cardiac intensive care units and 17% in elderly individuals admitted for acute cardiac conditions [51, 52]. Delirium is associated with mortality and bleeding events and up to 7% of patients >80 years with ACS develop delirium, with higher rates in comorbid and fragile patients [53].

The Comprehensive Geriatric Assessment (CGA) is a multidimensional, interdisciplinary diagnostic process to determine the medical, psychological and functional capacities of advanced-age patients, that enables the elaboration of a personalized integrated and coordinated plan for diagnosis, treatment, and follow-up [54]. Its use has been shown to increase survival and home stay after hospital admission in elderly patients [54].

## 6. Coronary Artery Disease Peculiarities of Octogenarians

Compared to younger patients, octogenarians more frequently present with multivessel disease [55, 56] and revascularization of the culprit lesion is less frequently achieved, in relation to the higher prevalence of calcification and tortuosity, complex lesions, need for bifurcation stenting, and ostial lesions [55, 56, 57]. In addition, the decision of whether to achieve complete revascularization or restraining the treatment of the culprit lesion remains a clinical challenge. Although the benefits of complete revascularization seem clearer in younger patients, there is still conflicting evidence regarding the elderly [58]. Multivessel percutaneous intervention seems to have better outcomes than culprit-only revascularization [59]. Coronary imaging and physiology are increasingly used to guide revascularization [60]. The recently published Functional versus Culprit-only Revascularization in Elderly Patients with Myocardial Infarction and Multivessel Disease (FIRE) trial, although done in the different context of ST-segment elevation, suggests a benefit for physiology-guided complete revascularization also in patients >75 years [61]. However, target lesion failure and bleeding complications are higher in the elderly, who seem to have a similar incidence of recurrent revascularization and stent thrombosis [57]. Other complications like contrast-induced nephropathy, acute heart failure and new-onset atrial fibrillation are also common in octogenarians.

Although no clear differences have been encountered between revascularization strategies, a less aggressive percutaneous intervention seems reasonable in old and fragile patients to avoid surgical-related complications [56, 62]. The radial artery approach is feasible and can be used in a similar way as in younger patients [57].

In summary, percutaneous interventions seem safe in octogenarians, with about 10% procedure complications, but all-cause death, cardiac mortality and recurrent myocardial infarction remain high in this population [57]. Preprocedural renal impairment and left ventricular systolic dysfunction are predictive of cardiovascular events [63].

## 7. Antithrombotic Treatment 

Age is included in most thrombotic and bleeding risk scales as a risk factor, and recent studies consider age to be a stronger predictor of bleeding than ischemic events [64]. Comorbidities have also been related to an increased bleeding risk, as well as frailty although to a lesser extent [65]. Some strategies to minimize bleeding risk in older patients include blood pressure control, gastroprotection, appropriate revascularization criteria, avoidance of pretreatment with purinergic receptor P2Y, G-protein coupled, 12 protein (P2Y12) inhibitors for NSTEMI, radial arterial access, and modulation of dual antiplatelet therapy duration [66]. Current guidelines recommend antithrombotic treatment with prasugrel in preference to ticagrelor or clopidogrel [67]. However, prasugrel is not recommended in patients >75 years old due to its results compared with clopidogrel in patients undergoing percutaneous coronary intervention for the ST-elevation myocardial infarction (TRITON-TIMI 38) trial, which showed a higher risk of fatal and life-threatening bleedings compared to clopidogrel in this different population with ST-segment elevation [68]. In addition, a progressive increase in the use of ticagrelor in older patients has been observed [69]. However, dual antiplatelet therapy with clopidogrel is still the most frequent combination among octogenarians [70, 71, 72, 73, 74]. In LONGEVO-SCA only 15% of octogenarians were treated with ticagrelor [75].

In a sub-analysis of the Platelet Inhibition and Patient Outcomes (PLATO) study comparing clinical outcomes in the elderly, ticagrelor reduced ischemia and mortality outcomes compared with clopidogrel without increasing bleeding risk [76]. However, in the Clopidogrel versus Ticagrelor or Prasugrel in Patients Aged 70 Years or Older with Non-ST-Elevation Acute Coronary Syndrome (POPular-AGE) trial that randomized clopidogrel versus ticagrelor or prasugrel, clopidogrel was not inferior to ticagrelor for all-cause death, myocardial infarction, stroke and minor bleeding, with a lower incidence of bleeding [77]. In addition, the Swedish Web-System for Enhancement and Development of Evidence-Based Care in Heart Disease Evaluated According to Recommended Therapies (SWEDEHEART) registry found an increased bleeding risk of ticagrelor compared to clopidogrel in patients ≥80 years with myocardial infarction [78].

There is some evidence for prasugrel dose reduction from 10 to 5 mg daily as an option in the elderly, with recent data showing comparable efficacy and safety to clopidogrel in patients >75 years [79, 80]. A meta-analysis that compared clopidogrel, prasugrel, and ticagrelor in older adults with ACS, clopidogrel seems to have the most favorable profile for reducing bleeding events [81].

Current guidelines recommend extending dual antiplatelet therapy beyond 1 year in patients with high ischemic risk. This strategy has been shown to be feasible in older patients, although their benefits seem to be attenuated in this population and therefore the implementation of extended dual antiplatelet therapy should be carefully evaluated [66, 82].

## 8. Risk/Benefit of the Two Strategies 

The management of NSTEMI in robust octogenarians should not differ from the one done in younger patients and an invasive strategy is the preferred option [2]. However, the approach to NSTEMI in octogenarians with severe or multiple comorbidities, those with dependence, frailty and/or limited life expectancy is not straightforward and conservative management is frequently an acceptable option [2, 83]. Invasive strategy reduces the risk of composite ischemic endpoints, particularly in high-risk patients but might produce complications [67]. The European Society of Cardiology guidelines recommend a routine invasive strategy for all patients irrespective of age, except for those deemed to be at a very low risk, where a selective invasive strategy is also accepted [67] but a global geriatric assessment might be a reason to change this approach in some octogenarians.

## 9. Strategy Trials 

The evidence supporting invasive strategy in the general population comes mainly from three ACS trials: FRagmin and Fast Revascularisation during InStability in Coronary artery disease (FRISC) II, RITA-3, and Invasive versus Conservative Treatment in Unstable Coronary Syndromes (ICTUS) [84]. FRISC II [84] randomized 2457 patients (461 >75 years). After 6 months, the composite outcome of death or myocardial infarction was lower in the invasive group (9% vs 12%). However, at five years of follow-up, mortality benefit seemed to vanish in patients >70 years [85]. RITA-3 trial included 1810 patients (226 >75 years) [86]. The combined outcome of death or myocardial infarction at 1 year was similar in both groups (8%), with angina improvement in the invasive arm. The benefit remained up until the 5-year follow-up, particularly in high-risk patients [9]. ICTUS randomized 1200 patients (152 >80 years), without significant differences in one-year endpoint of death, non-fatal MI, or rehospitalization (23% vs 21%) [87]. A metanalysis of these three trials showed that invasive strategy was associated with a reduction in the main outcome (5-year cardiovascular death or myocardial infarction) in patients aged ≥75 years (hazard ratio 0.71, 95% confidence interval 0.55 to 0.91) [88].

## 10. Strategy Trials in the Elderly 

A summary of the main randomized trials in advanced-age patients is shown in Table 1 (Ref. [89, 90, 91, 92, 93, 94, 95, 96]). Bach *et al*. [89] found that an invasive strategy was associated with a reduction in death or myocardial infarction at 6 months in the subgroup of patients >75 years, with a higher incidence of major bleeding. However, no differences in mortality or primary outcome have been described [90, 91]. The After-Eighty study [92] suggested an invasive strategy benefit regarding myocardial infarction and the need for urgent revascularization, that reduced with age, being non-significant in nonagenarians. However, nonagenarians seem to have a similar event rate to octogenarians [63]. Severe chronic kidney disease or left systolic dysfunction are strong predictors of outcomes that are frequently underrepresented [63]. In a meta-analysis of 20,540 patients aged >75 years, routine invasive therapy reduced mortality, myocardial infarction and stroke compared to conservative therapy, at the expense of an increase in major bleeding [93]. Another metanalysis of 13 studies in patients >75 years showed the benefit of an invasive strategy in long-term mortality, although the benefit was mainly driven by observational studies [94] and a higher risk of bleeding was shown for the invasive treatment group. Finally, another recent meta-analysis suggested that an invasive strategy was beneficial in terms of reduction of the need for revascularization in patients >65 years, with no significant effect on all-cause mortality, cardiovascular mortality, or myocardial infarction [95].

**Table 1.** **Trials comparing invasive with conservative strategy in non-ST-segment elevation acute myocardial infarction in the elderly**.

First author, year	Age (years), mean	N	Primary outcome	Benefits of invasive strategy
Bach 2004 [89]	>65 (72.9)	962	Mortality, nonfatal MI, rehospitalization, stroke and hemorrhagic complications	6-months death/MI: 10.8% vs. 21.6%
Savonitto 2012 [90]	>75 (81.8)	313	Composite of death, MI, disabling stroke, and repeat hospitalization for cardiovascular causes or severe bleeding	NS
Sanchis 2016 [91]	>70 (82.0)	106	Composite of all-cause mortality, reinfarction, and readmission for cardiac cause	NS
Tegne 2016 [92]	>80 (84.8)	457	Composite of MI, need for urgent revascularization, stroke, and death	40.6% vs. 61.4%, HR = 0.53
Gnanenthiran 2017 [93]	>75	20,540	In-hospital mortality, mortality at follow-up, MI, revascularization, rehospitalization for cardiac causes, stroke, major bleeding	Inhospital mortality OR = 0.65, 95% CI: 0.53–0.79
Ma 2018 [94]	>75	832,007	Death at follow-up from 6 months to 5 years	RR = 0.65, 95% CI: 0.59–0.73
Reaño 2020 [95]	>65	3768	All-cause mortality, cardiovascular mortality, MI, stroke, need for revascularization, recurrent angina.	NS
Sanchis 2023 [96]	>70 (86.0)	167	Number of days alive and out of the hospital.	NS

MI, miocardial infarction; NS, non significant; HR, hazard ratio; OR, odds ratio; RR, risk ratio; CI, confidence interval.

## 11. Strategy Trials in Frail Patients

Observational studies suggest that an invasive strategy benefit persists in the elderly but there are conflicting data regarding whether the benefit persists or is even higher in frail patients [97, 98, 99]. Randomized trials have failed to show an invasive strategy benefit in frail patients. The Randomized comparison between the invasive and conservative strategies in comorbid elderly patients with non-ST elevation myocardial infarction (MOSCA) trial included patients with >70 years and more than two comorbidities and found no differences in the primary outcomes of all-cause mortality, reinfarction and admission for cardiac cause [91]. The more recent Invasive Versus Conservative Strategy in Frail Patients With NSTEMI (MOSCA-FRAIL) included patients >70 years with frailty (Punctuating >4 in the Clinical Frailty Scale). There were no differences in the primary outcome of days alive outside the hospital in the 1-year follow-up, with a trend to a benefit for conservative strategy [96]. The British Heart Foundation Older Patients With Non-ST SEgmeNt elevatIOn myocaRdial Infarction Randomized Interventional TreAtment Trial (SENIOR-RITA) in frail patients >75 years is still ongoing [100].

## 12. Patients’ Perspectives and Decision-Making 

Patients’ perspectives, values and opinions are key in the decision-making process and should be incorporated into the protocolized decision [101]. Some patients might prefer the option with the lowest risk for harm, even if the long-term benefit is lower [102]. In addition, expected life expectancy should be one of the variables that is incorporated in the equation, as conservative management is probably a better option in patients with life expectancies <1–2 years.

## 13. Conclusions

NSTEMI is one of the main causes of mortality and morbidity in the elderly. Age alone should not be the main factor to consider when offering an invasive strategy, as the heterogeneity of this population makes it imperative for clinicians to assess other variables like frailty, comorbidities, and other geriatric conditions. The risk of futility needs to be taken into account and an invasive approach should not be a static choice, as some elderly patients might benefit from an initial conservative approach open to subsequent invasive management depending on the clinical course (recurrent angina, ventricular arrhythmias, heart failure). Further evidence, ideally from prospective randomized clinical trials is urgent, as the population keeps growing.
